# Assessment of laparotomy-induced stress response in opium- and morphine-addicted rats by measuring serum glucose and corticosterone levels: an animal experiment

**DOI:** 10.1097/MS9.0000000000003010

**Published:** 2025-02-06

**Authors:** Yeganeh Pakbaz, Sepideh Eydivandi, Ayda Khandani, Mohammad Poursalehian, Hani Ghadri, Massoud Baghai Wadji

**Affiliations:** a Breast Health & Cancer Research Center, School of Medicine, Iran University of Medical Sciences, Tehran, Iran; b Gastrointestinal and Liver Diseases Research Center, School of Medicine, Iran University of Medical Sciences, Tehran, Iran; c Men’s Health and Reproductive Health Research Center, Shahid Beheshti University of Medical Sciences, Tehran, Iran; d Students’ Scientific Research Center, Tehran University of Medical Sciences, Tehran, Iran; e Student Research Committee, School of Medicine, Iran, University of Medical Sciences, Tehran, Iran; f Department of Surgery, Iran University of Medical Sciences, Firoozgar Hospital, Tehran, Iran

**Keywords:** animal study, laparotomy, morphine, opium, rat, surgical stress response

## Abstract

**Background:**

Surgical procedures induce stress responses similar to severe illnesses, activating the metabolic and neuroendocrine systems, especially the hypothalamic–pituitary–adrenal (HPA) axis. The resulting cortisol surge maintains homeostasis but can adversely affect recovery by elevating blood glucose levels and increasing the risk of complications. Given the high prevalence of opium use, especially in the Middle East and southwestern Asia, and its suspected impact on HPA axis function, this study assesses corticosterone (CS) and glucose as indicators of impaired neuroendocrine responses to surgical stress in an animal model with chronic opioid use.

**Materials and methods:**

Thirty-six male Wistar rats were randomly assigned to three groups: morphine-addicted, opium-addicted, and control. Addiction was induced by administering increasing doses of morphine or opium in drinking water, as verified by naloxone injections. Laparotomy was performed under ketamine and xylazine anesthesia. Blood samples were collected post-surgery and post-recovery to measure the CS and glucose levels.

**Results:**

This study included 30 rats, with 10 rats per group. Post-surgery, mean CS levels were higher in the control group compared to the addicted groups, although not significantly higher. Thirty minutes post-recovery, CS levels remained elevated in the addicted groups. Mean glucose levels were significantly higher in the control group both immediately and 30 minutes post-recovery, indicating a sustained hyperglycemic response. No significant differences were observed between addicted groups in glucose levels.

**Conclusion:**

Our study suggests that chronic opioid use may reduce the neuroendocrine response to surgical stress, as shown by lower CS levels in the addicted rats. This aligns with existing research on opioids and stress responses. However, the small sample size and lack of baseline measurements limit the findings. Future studies should use larger, more diverse samples and additional biomarkers. This pilot study highlights the need for further research on altered stress responses in opioid-addicted patients undergoing surgery, emphasizing the importance of tailored postoperative care.

## Introduction

Surgical procedures can induce stress levels in patients comparable to those caused by severe illness, trauma, and burns[1]. In response to this stress, the body activates both metabolic and neuroendocrine systems^[2-4]^. One of these responses involves activation of the hypothalamic–pituitary–adrenal (HPA) axis. Major surgeries primarily stimulate the HPA axis, causing the hypothalamus to secrete corticotropin-releasing hormone (CRH). CRH then prompts the pituitary gland to release adrenocorticotropic hormone (ACTH), which subsequently stimulates the adrenal cortex to produce cortisol[5].

The HPA axis plays a crucial role in maintaining homeostasis, producing cortisol in humans and corticosterone (CS) in rats, both of which are essential for managing stress responses and other biological processes^[4,6]^. Cortisol, in particular, induces gluconeogenesis, leading to increased blood glucose levels^[1,7]^. Although hyperglycemia is necessary for maintaining bodily homeostasis during stress, it can also have detrimental effects^[8,9]^. Elevated cortisol levels can impair wound healing and increase the risk of complications such as ischemia, sepsis, infections, and even mortality[1]. This dual impact of cortisol-induced hyperglycemia underscores the complexity of the body’s response to stress and highlights the need for careful management in different clinical settings^[7,10]^.

**HIGHLIGHTS**

- Elevated cortisol can impair wound healing and increase complications.
- Opium addiction may alter neuroendocrine responses to surgical stress.
- The control group showed significantly higher post-surgery glucose levels.
- No significant difference in CS levels between the addicted and control groups.
- Comprehensive post-op strategies should address the impact of chronic opioid use.

Opium, containing various alkaloids, including morphine, is used both therapeutically and illicitly in over 50 countries worldwide[11]. The prevalence of opium use is particularly high (2.6%) in the Middle East and southwestern Asia[12]. Recent estimates from 2016 indicate that opioid addiction has a significant impact in Iran, affecting nearly 2.1% of the population, equivalent to approximately 1.12 million adults[13]. Long-term opioid addiction is suspected to impair HPA axis function, potentially leading to an impaired physiologic response to stress. This response can arise from adrenal disease, ACTH deficiency, or suppression of ACTH due to opioid drugs^[14-16]^.

Given the prevalence of opium addiction and its potential impact on surgical outcomes, this study aims to assess CS and glucose levels as indicators of impaired neuroendocrine responses to surgical stress in an animal model after surgery and recovery. This will help assess the impact of surgical stress on postoperative outcomes. This research aims to fill the knowledge gap by examining the physiological (metabolic and neuroendocrine) responses to surgical stress and recovery in the context of chronic opioid use.

## Methods and materials

### Animals

A total of 36 male Wistar rats weighing approximately 220–250 g at the beginning of the experiment and around 8 weeks old were obtained from the Pasteur Institute of Iran. These rats were naive and were used exclusively for this study. They were transferred to the animal lab of the Kerman Neuroscience Research Center. The rats were kept in pairs in wire cages measuring 30 × 40 × 50 cm and placed in a controlled environment featuring a 12-hour light–dark cycle with temperatures maintained at 23 ± 5°C for 26 days. Cages were cleaned every 3 days. Throughout the study, the rats were provided with both food and water *ad libitum*.

### Experimental design

The rats were randomly assigned to one of 3 groups of 12: a morphine-addicted group, an opium-addicted group, and a control group. Additionally, two rats from each addicted group received naloxone injections to confirm addiction. Two rats from the control group were initially included but were later excluded due to the potential risk of mortality associated with naloxone injections. All experiments were conducted between 8:00 a.m. and 2:00 p.m. to minimize the effects of circadian rhythm on the experimental outcomes[17]. All the allocation stages and the experiments were performed in a blinded manner. Addiction was induced in the rats by gradually increasing doses of the substance administered in their drinking water, following a specific protocol outlined below.

### Addiction induction procedure

#### Morphine addiction induction

Rats in the morphine group received morphine sulfate powder (Temad, Tehran, Iran) dissolved in their drinking water beginning 26 days before the surgery. To mitigate the bitterness of morphine, a 5% sucrose solution was added to the water[18]. Initially, for the first 48 hours, 0.1 mg of morphine sulfate per milliliter of drinking water was provided to the animals. Subsequently, this dosage was gradually increased to 0.2, 0.3, and 0.4 mg/ml over subsequent 48-hour periods. This dosage regimen was maintained until the conclusion of the 26 days. To confirm the addiction status of the rats, following cessation of the drug administration period, a subset of rats (two rats) was randomly administered intraperitoneal injections of naloxone (2 mg/kg). Withdrawal symptoms, including muscle twitching and diarrhea, were observed 30 minutes post-injection; these symptoms verified the addiction[19].

#### Opium addiction induction

To induce opium addiction in rats, a protocol similar to that used for morphine addiction was employed. Opium, containing approximately 16% morphine[20], sourced from the Kerman Neuroscience Research Center, was dissolved in the drinking water of the rats over four 48-hour periods using the same method. The dose started at 0.625 mg of opium per milliliter of drinking water and gradually increased to 1.25, 1.87, and finally to 2.5 mg/ml over subsequent 48-hour intervals. The concentration of opium remained constant until the conclusion of the 26th day. Boiling water was used to dissolve the opium, and sucrose powder was added after cooling to mask its bitterness. A naloxone test similar to the one used in the morphine group confirmed addiction and ensured the presence of withdrawal symptoms before the study’s inclusion. Following the confirmation of addiction, we excluded six rats from the study and initiated the experiments.

### Anesthesia and surgical procedures

#### Anesthesia induction

All rats received intraperitoneal injections of ketamine (60 mg/kg, Ketalar^®^; Pfizer, Netherlands) along with xylazine (5 mg/kg, Rompun^®^; Bayer, Germany) 15 minutes before starting the surgical procedure^[21,22]^. The anesthesia lasted approximately 45 minutes.

#### Surgery

Laparotomy surgery with parietal peritoneum stimulation was performed on all the rats after confirming their unconscious state, verified by eliciting a response to painful stimulation applied to their paws. First, the abdominal area of each rat was disinfected with Betadine—povidone-iodine 10% (BETADINE^®^ Solution, toluidine 10% 1 L solution, Tolid Daru Company, Iran), shaved, and a 3–4 cm longitudinal incision was made below the xiphoid process along the midline of the abdomen using a #10 surgical blade. The skin, fascia, and abdominal muscles were dissected, and the surrounding fat was removed to expose the intestines. The parietal peritoneal stimulus stress was induced by gently manipulating the intestines with sterile gauze. Subsequently, the layers of the abdomen, muscles, and skin were sutured using a 4-0 nylon thread.

### Blood sample collection and analysis

#### Blood sample collection

After surgery, a whole blood sample was obtained from the retro-orbital sinus plexus for the analysis. The rats were placed under a heating device to maintain their central body temperature and prevent hypothermic stress. Thirty minutes after regaining consciousness, a second whole blood sample was obtained from the jugular vein of each rat as the post-recovery sample.

#### Blood sample analysis

**Figure 1.**
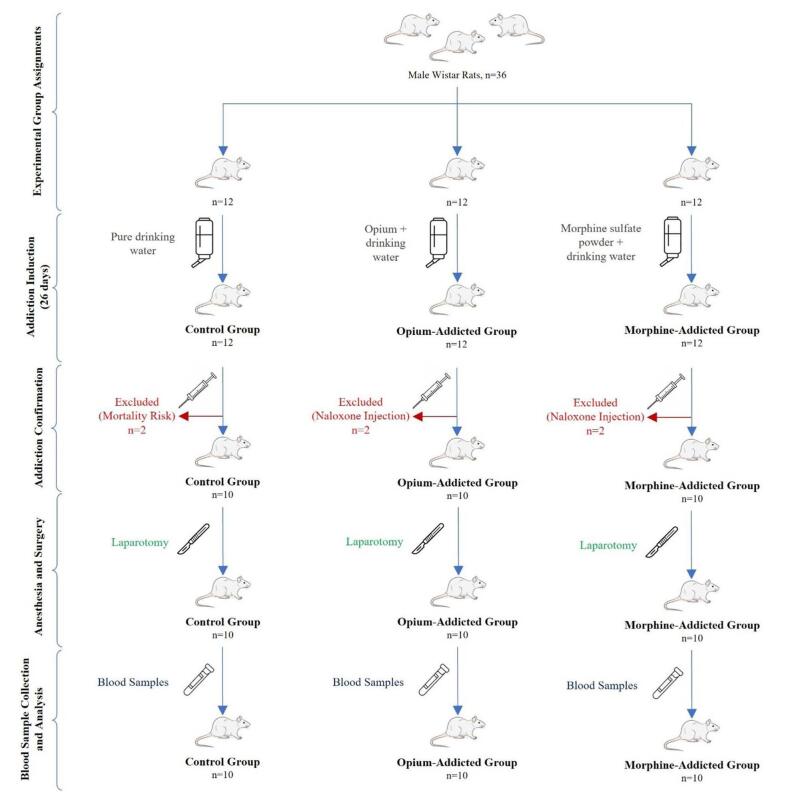
Study design of experimental groups and procedures.

After collecting the blood samples, they were transferred into glass test tubes soaked in a solution of 5% ethylenediaminetetraacetic acid and immediately centrifuged for 15 minutes at 3000 rpm (rounds per minute). The plasma was separated using manual pipettes and emptied into a microtube kept in a freezer at −70°C and then sent to the central laboratory to be measured for glucose and CS levels. The plasma concentration of CS was assessed using an enzyme-linked immunosorbent assay (ELISA) test with an ELISA kit obtained from Glory Science Company, based in Hong Kong, China (Fig. 1).

### Statistical analysis methods

Statistical analyses were conducted using SPSS version 22.0 software. Descriptive data were presented as the mean ± SD (range). The Kruskal–Wallis test was employed to compare the levels of CS and glucose at different time points, and results with a *P*-value of 0.05 or less were considered statistically significant. The Dunn’s test was employed as a *post hoc* test.

This work has been reported in line with the ARRIVE criteria[23].

## Results

The study included 30 male Wistar rats, with 10 rats per group. The average weight of the rats increased significantly from 241.30 ± 15.98 to 245.13 ± 15.59 g over 26 days (Table 1).

**Table 1:** Evaluation of the weights of rats in all groups.

Stage	Group	Sample size (*n*)	Mean weight ± SD	Minimum	Maximum
Day 1	Control	10	234.70 ± 15.19	205	257
	Morphine-addicted	10	245.10 ± 13.94	223	267
	Opium-addicted	10	243.60 ± 17.85	220	270
	Total	30	241.30 ± 15.89	205	270
Day 26	Control	10	248.60 ± 17.55	220	277
	Morphine-addicted	10	241.80 ± 17.64	230	260
	Opium-addicted	10	245.00 ± 11.79	215	272
	Total	30	245.13 ± 15.59	215	277

SD, standard deviation.

### CS levels

There were no significant differences in the mean CS levels among the three groups post-surgery and post-recovery (*P* > 0.05) (Table 2). Although their difference was not significant, CS levels were slightly higher in the control group immediately after surgery. Thirty minutes after recovery, the CS level of the control group dropped while the CS level of the addicted group remained high (Table 3).

**Table 2:** Comparison of CS and glucose levels among all groups.

Stage	Group	Mean ± SE	*P-*value
CS levels			
Post-surgery	Control	76.60 ± 5.59	0.675
	Morphine-addicted	71.40 ± 4.40	
	Opium-addicted	69.50 ± 3.94	
Post-recovery	Control	58.40 ± 4.89	0.388
	Morphine-addicted	70.70 ± 9.10	
	Opium-addicted	66.00 ± 4.56	
Glucose levels			
Post-surgery	Control	241.80 ± 8.43	<0.001
	Morphine-addicted	193.40 ± 11.29	
	Opium-addicted	153.80 ± 14.47	
Post-recovery	Control	280.10 ± 33.38	0.038
	Morphine-addicted	188.50 ± 24.59	
	Opium-addicted	191.50 ± 43.69	

CS, corticosterone; SE, standard error.

**Table 3:** Change in CS and glucose levels among all groups.

Group	Mean ± SD	*P*-value
Change in CS levels		
Control	−18.20 ± 8.92	0.316
Morphine-addicted	−0.70 ± 10.16	
Opium-addicted	−3.50 ± 6.43	
Change in glucose levels		
Control	38.30 ± 33.23	0.602
Morphine-addicted	−4.90 ± 22.37	
Opium-addicted	37.70 ± 45.23	

CS, corticosterone; SD, standard deviation.

### Glucose levels

Significant differences in mean glucose levels were observed post-surgery and post-recovery among the three groups (Table 2). Glucose levels were higher in the control group immediately after surgery (*P* < 0.001). Glucose levels remained higher in the control group 30 minutes after recovery (*P* = 0.038). Their respective changes are detailed in Table 3.

## Discussion

**Figure 2.**
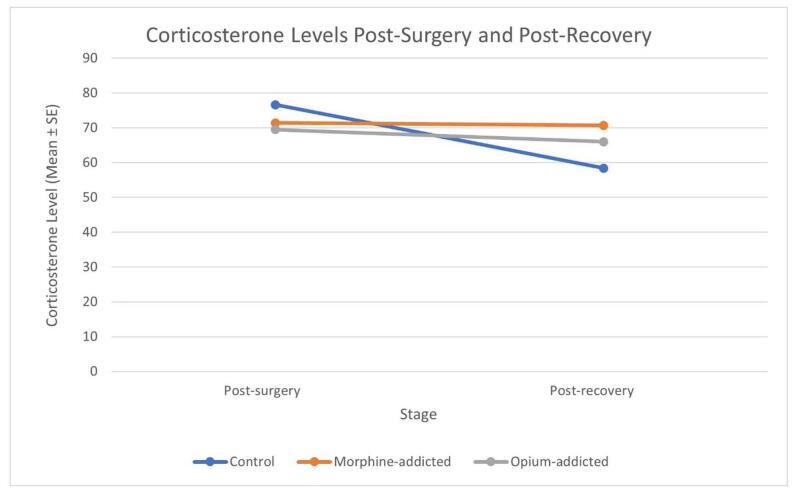
Mean CS levels post-surgery and post-recovery.

**Figure 3.**
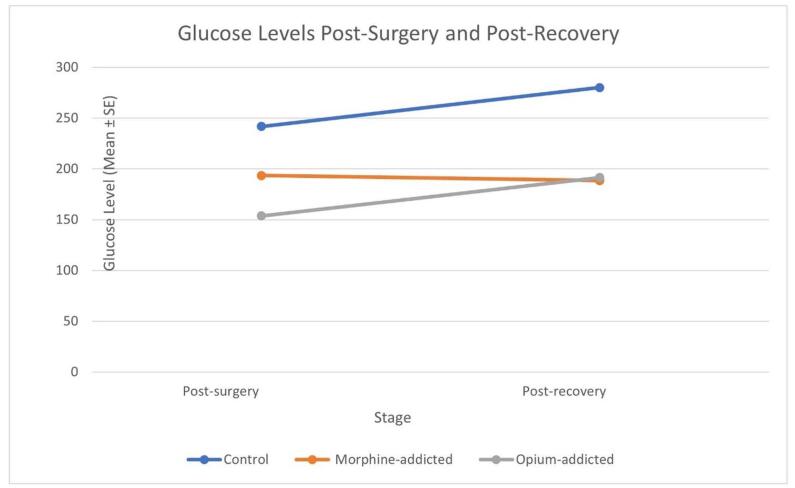
Mean glucose levels post-surgery and post-recovery.

Several studies consistently demonstrate that opium-addicted surgical patients experience significantly higher postoperative morbidity and mortality, longer hospital stays, an increased need for secondary surgeries, higher 30-day readmission rates, and elevated health care costs^[24-27]^. Furthermore, our extensive experience in postoperative care for opium-addicted patients, especially in Iran’s Kerman Province, has revealed distinct surgical responses compared to non-addicted individuals. This prompted us to investigate whether addicted patients might have altered metabolic and neuroendocrine responses to stress. Specifically, we focused on investigating the HPA axis, a critical component of the stress response system. Our study found that after laparotomy, the mean CS levels were lower in the opium- and morphine-addicted groups compared to the control group. However, no statistically significant difference was observed in CS levels between the three groups (Fig. 2). The mean serum glucose level in the control group was significantly higher than in the addicted groups in both post-surgery and post-recovery stages. There was not a notable difference in mean serum glucose levels between the morphine-addicted and opium-addicted groups (Fig. 3).

Buckingham and Cooper measured post-surgical ACTH and serum CS levels in male Sprague-Dawley rats receiving both acute and chronic morphine treatment following laparotomy. They found that chronically morphine-treated rats showed no stress response, consistent with our findings[28].

Additionally, Yarigholi *et al*[29] conducted a study on 25 male Wistar rats to assess the effect of cirrhosis on the HPA axis during surgery in cirrhotic rats by measuring changes in serum CS and blood sugar levels before, immediately after, and 30 minutes post-surgery. Similar to the chronic administration of opium and morphine^[28,30,31]^, cirrhosis was associated with adrenal insufficiency^[32,33]^. They observed a noticeable increase in serum CS levels during three-time levels of surgery; however, this increase was significantly lower than that observed in the control group. Additionally, the mean blood glucose level was higher in the control group compared to the cirrhotic rats.

While research suggests that opiates affect glucose homeostasis, the precise mechanisms remain unclear. Opioids exert complex effects on various hormones of the endocrine system in both humans and animals, often varying depending on whether exposure is acute or chronic[34].

We consider this study to be a pilot study to evaluate the response to surgical stress in opium- and morphine-addicted surgical rats. A potential limitation of this study is the lack of baseline serum samples from rats before surgery, which prevents us from establishing baseline CS levels for comparison with post-surgery levels. This limitation impacts our ability to accurately assess the effects of chronic opioid use on the stress response. Furthermore, the small sample size reduces the statistical power of our findings; increasing the sample size would improve the reliability and robustness of the data. Another limitation is the inclusion of only male rats, which restricts the applicability of our findings to females. Moreover, as an animal study, some inherent species differences limit the direct application of our results to human subjects. Additionally, measuring additional biomarkers such as IL-6, C-reactive protein, and white blood cell count could provide more comprehensive insights into the inflammatory response following surgery. We also acknowledge that our study lacks long-term postoperative monitoring, which limits our ability to assess sustained neuroendocrine changes over time. Additionally, uncontrolled factors in the lab environment, such as handling stress, may influence CS and glucose levels, introducing variability into the results.

In conclusion, this study suggests that opium-addicted rats are more likely to exhibit an abnormal neuroendocrine response to surgical stress. More scientific research is needed to fully understand the mechanisms underlying these altered stress responses. Future studies should focus on elucidating the specific pathways involved and the potential long-term implications for human patients. Additionally, exploring potential interventions or treatment modifications for opium-addicted patients undergoing surgery could provide significant clinical benefits. These findings emphasize the importance of considering addiction status in postoperative care, as well as the need for a multidisciplinary approach to optimize outcomes for these vulnerable patients.

## Data Availability

The data that support the findings of this study are not publicly available due to institutional or legal restrictions concerning animal research but are available from the corresponding author [M.B.W.] upon reasonable request.

## Assistance with the study

The authors would like to express their sincere gratitude to the Kerman Neuroscience Research Center for providing access to the animal laboratory, which was essential for the successful completion of this study. We also thank them for their expertise and support before and during the experiment.
